# Ontogeny of Mouse Vestibulo-Ocular Reflex Following Genetic or Environmental Alteration of Gravity Sensing

**DOI:** 10.1371/journal.pone.0040414

**Published:** 2012-07-10

**Authors:** Mathieu Beraneck, Mickael Bojados, Anne Le Séac’h, Marc Jamon, Pierre-Paul Vidal

**Affiliations:** 1 CNRS UMR 8194, Université Paris Descartes, Sorbonne Paris Cité, Paris, France; 2 INSERM UMR 1106, Aix-Marseille Université, Marseille, France; Imperial College London, United Kingdom

## Abstract

The vestibular organs consist of complementary sensors: the semicircular canals detect rotations while the otoliths detect linear accelerations, including the constant pull of gravity. Several fundamental questions remain on how the vestibular system would develop and/or adapt to prolonged changes in gravity such as during long-term space journey. How do vestibular reflexes develop if the appropriate assembly of otoliths and semi-circular canals is perturbed? The aim of present work was to evaluate the role of gravity sensing during ontogeny of the vestibular system. In otoconia-deficient mice (*ied*), gravity cannot be sensed and therefore maculo-ocular reflexes (MOR) were absent. While canals-related reflexes were present, the *ied* deficit also led to the abnormal spatial tuning of the horizontal angular canal-related VOR. To identify putative otolith-related critical periods, normal *C57Bl/6J* mice were subjected to 2G hypergravity by chronic centrifugation during different periods of development or adulthood (*Adult-HG)* and compared to non-centrifuged (*control*) *C57Bl/6J* mice. Mice exposed to hypergravity during development had completely normal vestibulo-ocular reflexes 6 months after end of centrifugation. *Adult-HG* mice all displayed major abnormalities in maculo-ocular reflexe one month after return to normal gravity. During the next 5 months, adaptation to normal gravity occurred in half of the individuals. In summary, genetic suppression of gravity sensing indicated that otolith-related signals might be necessary to ensure proper functioning of canal-related vestibular reflexes. On the other hand, exposure to hypergravity during development was not sufficient to modify durably motor behaviour. Hence, 2G centrifugation during development revealed no otolith-specific critical period.

## Introduction

The vestibular organs consist of complementary sensors: the semicircular canals detect angular accelerations while the otoliths detect linear accelerations, including the constant pull of gravity. Integration by the vestibulo-cerebellum of canal and otolith information to visual and proprioceptive inputs generates an estimate of the position and movements of the head in space [1]. In frog, most of the central vestibular neurons receive convergent monosynaptic inputs from a single semicircular canal and from the spatially complementary otolith [2]. In rat, convergence of macular and canal inputs concern about 80% of tested vestibular neurons [3]. In cat, 1/3 of studied neurons receive convergent inputs from vertical canals and otoliths and 1/5 receive convergent inputs from horizontal canals and otoliths [4]. The anatomical convergence and functional complementarities between canals and otoliths raise questions on how spatial refinements of vestibular microcircuits at the origin of the vestibulo-ocular reflex (VOR) occur during development and adapt throughout lifespan. As for other developing systems, ontogeny of the vestibular system depends on both genetics and experience [5]. Initial formation, pathfinding to specific targets and survival of inner ear neurons are based initially on genetic programs [6] during the 2 perinatal weeks [7]. Along the 3-neurons-arc that drives the VOR, connectivity is established around birth independently of vestibular activity [8]. Then, late activity-mediated maturation would refine its function [9], [10].

In that context, various experiments investigated the effects of an environmental alteration of gravity on the development of the vestibular system. Prenatal exposure of rats to weightlessness induced severe and often long lasting abnormalities on vestibular-related behaviors [7]. Also, increasing gravity during development induced non-lasting effects on the morphology and physiology of the vestibular periphery [11]–[13] however correlated to limited locomotor deficits [14]–[16]. Overall, a change in gravity-load for periods including development would modify both peripheral and central vestibular-related circuits, but the persistence of these alterations remains uncertain [7], [17] and the ontogeny of the oculomotor control was not examined.

Therefore, this study was aimed at investigating the role of the gravito-inertial information in the combined development of otolith-based and canal-based vestibular reflexes. In particular, the two following hypotheses were tested: i) tuning of canal-related vestibulo-ocular reflexes (VOR) need gravito-inertial information and ii) alteration of gravity during critical periods of development indefinitely impairs the acquisition of otolith-specific reflex and of canal-specific VOR. To examine these questions, the VOR presents many experimental advantages. First, VOR circuitry has been investigated in detail. Second, although body movements are drastically modified under altered gravity due to the loading/unloading of large body segments, eyes are mechanistically less affected. Finally, otolith-specific reflex (e.g. maculo-ocular reflex) can be evaluated using paradigms such as the off-vertical axis rotation test [18].

In order to alter the sensing of gravito-inertial forces during development, we used two types of paradigms. First, otoconia-deficient mice, which suffer from the absence of gravity-related modulation of otolith inputs [19] were used as a model of development without the vestibular sensing of gravity [20]. Second, the effect of an increase of gravity-load on the gaze stabilization was studied in mice centrifuged during adulthood, during the entire development, or for periods covering respectively the early onset or late maturation of the vestibular ontogeny [7].

## Methods

### Ethics Statement

All procedures used were in strict compliance with the European Directive 86/609/EEC on the protection of animals used for experimental purposes. CNRS review board and the Direction Departementale des Services Vétérinaires specifically approved this study; authorization number 75–1641.

Forty-six normal mice (*C57Bl/6J* - Charles River Laboratories, France), including 31 mice born and raised in 2G centrifuge were used in this study (see detailed procedure below). In addition, otoconia-deficient mice (inner ear defect, *ied*, see [20]) with a *C3HeB/FeJ* Background were initially purchased from the Centre De Transgénèse d’Orléans, France, and breeding was subsequently done in our institution. *Ied* mutation consists in a splicing defect on the chromosome 5 otop 1 gene, which leads to complete otoconial agenesis [20]–[22]. Thirteen *ied* mice aged 6 months were phenotyped during swimming task to ensure inner ear deficiency [23], [24]. As other otoconia-deficient strains, *ied* mice also presented a head deviation in frontal plane (or head-tilt, Figure S1; [20]).

### Centrifuge Configuration and Housing Conditions

Hypergravity was produced using a centrifuge consisting in 1.4 m radius carousel with gondolas hanged on the periphery (maximum radius during rotation: 1.8 m) [25]. 2G vector at the center of the gondolas (±5% on the edges) was produced with 29.6 rpm rotational speed. *C57Bl/6J* mice were placed in standard cages located in the gondolas. Cages were supplied enough food and water for 3 weeks and were left undisturbed during the chronic centrifugation. When centrifugation duration exceeded 3 weeks, centrifugation was interrupted during minimal time needed for refilling food and water. Infra-red camera videos fixed on the centrifuge arms allowed a remote day and night control of the mice in their cages. All the environmental variables, except the gravity level, were the same as in standard housing. The cages containing *control C57Bl/6J* mice were placed in similar gondolas and in the same room as the centrifuged mice.

### Description of the Groups of Centrifuged Mice

*C57Bl/6J* mice bred in the centrifuge had a previous experience of chronic centrifugation of at least eight weeks, and were all multiparous to reduce the risks of misbehavior against the pups during the centrifugation. The experiments involved four groups of *C57Bl/6J* mice centrifuged during different periods and duration of development or adulthood. For Developmental nomenclature “E10” indicates 10^th^ embryonic day of development and “P10” 10^th^ postnatal day of maturation.

Group *Control* (n = 8): *C57Bl/6J* mice were born and raised in the room of the centrifuge, but were never exposed to hypergravity (HG). Control mice were tested at age of 6 months.
Group *Adult-HG* (n = 7): *C57Bl/6J* mice born and raised in the centrifuge room were exposed to 50 days of 2G centrifugation between the age of 2–4 months. Mice were tested 1 month and 6 months after their return to normogravity.
Group *Pre* (E0–P10, prenatal exposition to HG; n = 8): *C57Bl/6J* mice were conceived and born in the centrifuge. The pups remained with their parents in the centrifuge until 10^th^ postnatal day. Mice were then put in standard housing under normal gravity in the same room.
Group *Post* (P10–P30: postnatal exposition to HG; n = 8): *C57Bl/6J* mice reproduced in standard housing. The pups were transferred to the centrifuge with their parents at 10^th^ postnatal day. Mice were left in the centrifuge until the 30^th^ postnatal day, and then returned to standard housing in the centrifuge room.
Group *Full* (E0–P30: complete development in HG; n = 15): The conception and delivery occurred in the centrifuge but the *C57Bl/6J* mice remained in the centrifuge until 30^th^ postnatal day.

The pups were weaned at the age of 30 days, they were weighed, individually tagged with RFID microchips and grouped 3 same sex mice per cage. They were left undisturbed until the age of 2 months when a set of behavioral tests [26] were carried out. Once the tests were completed, sets of mice were sacrificed for tissue analysis, while the other returned in standard housing for 5 months for a second series of tests.

### Surgeries

Surgical preparation and postoperative care for head implant surgery have been described previously [27]. Gas anesthesia was induced using Isoflurane. A small custom-built head holder was then cemented (C&B Metabond) to the skull just anterior to the lambda landmark [28]. Following the surgery, animals were isolated and closely surveyed for 48 hours. Buprenorphine (0.05 mg/kg) was provided for postoperative analgesia and care was taken to avoid hypothermia and dehydration.

### Vestibular Stimulations and Data Acquisition

Recorded eye and head position signals were sampled at 1 kHz, digitally recorded (CED power1401 MkII) under spike 2 environment and later exported into the Matlab (The MathWorks) programming environment for off-line analysis.

Mice were head-fixed at a ∼30° nose-down position to align the horizontal canals on yaw plane [29]–[31]. Because the precise orientation of the canals in *C3HeB/FeJ* background was never reported, we tested in a preliminary experiment the efficiency of the horizontal vestibulo-ocular reflex for different head-pitch angles (Figure S1, A) and found that a head pitch of 30° was about optimal. Accordingly, *ied* were tested following the same procedure as *C57Bl6/J* mice.

Mice were initially tested during sinusoidal rotations (0.2–2 Hz; 40°/s) in yaw plane. Rotations at constant velocities around vertical axis were also applied to *ied* and 50 days exposed groups (Full and Adult-HG groups) at 50°/s. Once yaw testing completed, the table was tilted 17° off-vertical axis with the mouse in a nose-down position. Off-vertical axis rotation (OVAR) responses were tested at constant velocities of 30 and 50°/s in randomized CW or CCW directions. For each paradigm at least 10 consecutives cycles were recorded.

### Eye Movements Measurements during OVAR

The experimental set-up, apparatus, and method of data acquisition used to record eye movements were similar to those previously described [27], [32]. Animals were placed in a custom built Plexiglas tube secured on the superstructure of a vestibular stimulator. Eye movements were recorded using an infrared video system (ETL-200, ISCAN, Burlington MA). As eyes were recorded in the absence of light, 2% pilocarpine (Laboratoire Chauvin) was applied to keep the pupil size constant [33], [34]. In *ied* mice, an ocular instability in dark was occasionally observed at the onset of the experiment just after the animal was head-fixed (Figure S1, A, left trace). Instability could consist in horizontal, vertical, and torsion cyclic movements. Importantly, the *ied* mice are not blind as the eye instability was reduced in light (Figure S1, A, middle and left trace). In cases where instability did reappear during the experiment, the data were only taken after it abated, either spontaneously or after the animal was transiently put back in light until stabilization.

**Figure 1:**
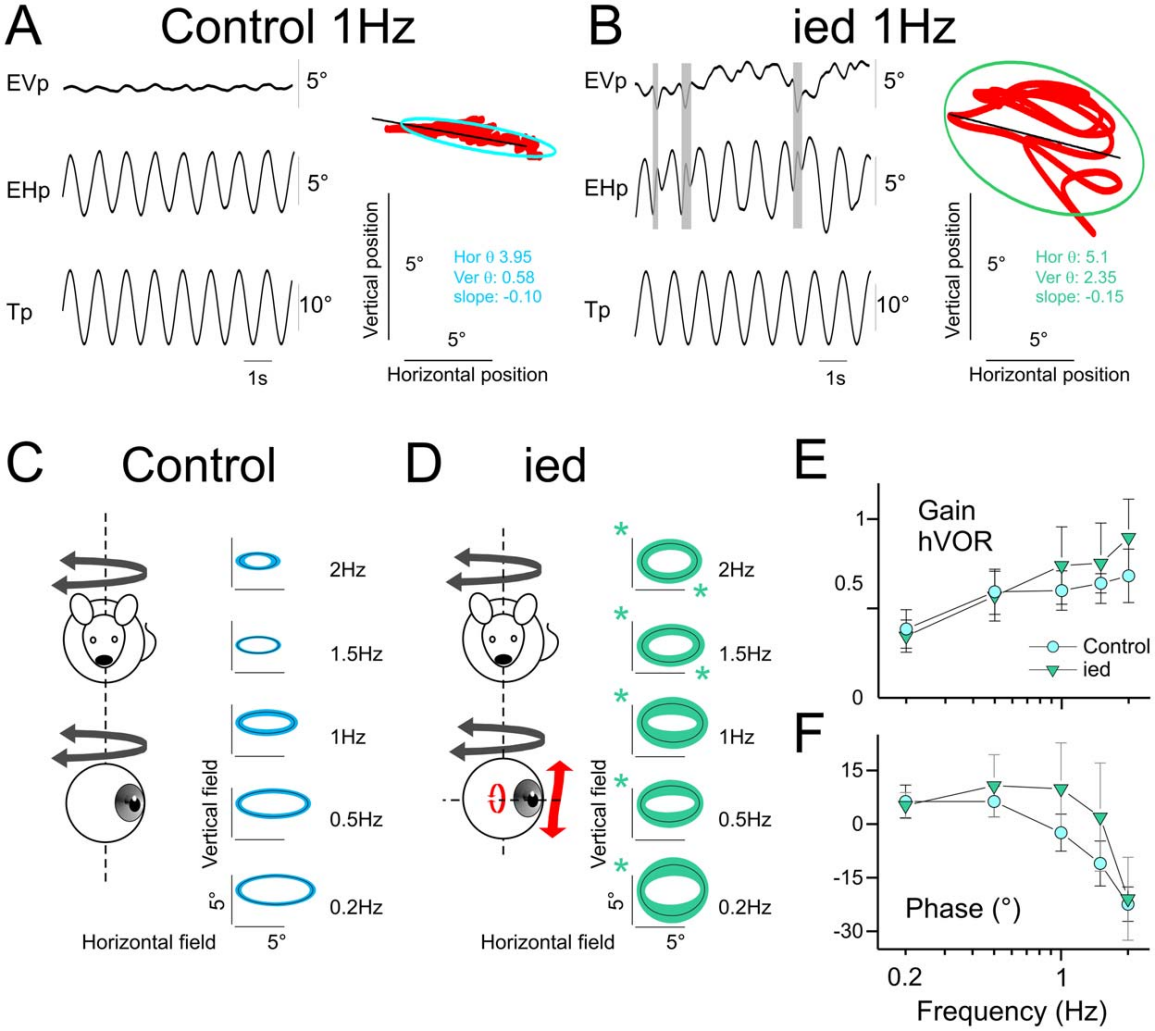
Horizontal angular vestibulo-ocular reflex in *C57Bl/6J* and *ied* mice. A–B, Example of eye movements evoked in *C57Bl/6J* (A) or *ied* (B) mice during 1 Hz sinusoidal oscillation in horizontal. Shaded areas indicate quick phases. Plots present oculomotor fields. Red points are the eye position of the same traces. The ellipses present 95% of the horizontal and vertical eye positions. θ are horizontal and vertical variance; inclination of the ellipse was computed as the slope of the linear regression between vertical and horizontal eye positions. C–D Averaged oculomotor fields in *C57Bl/6J* (C) and *ied* (D) mice across tested frequencies. Line and surface of the ellipses present the mean and standard deviation of the population, respectively. The slopes of the ellipses are the mean slope of the individuals’ ellipses. Green asterisks indicate significantly larger response in *ied* compared to *C57Bl/6J*. E–F, Gain (E) and timing (F) of the horizontal component of eye movement responses for *C57Bl/6J* and *ied* populations. Asterisk indicates statistical difference with *p*<0.05. Tp, Table position; EVp, Eye Vertical position; EHp, Eye Horizontal position. In this and following figures, plots present mean ± SD.

The measurements for transforming the 2D eye position into 3D coordinates is the most accurate when the eye is moving along a single axis [35]; see for discussion [31]. During OVAR stimulation the eye position however changes both horizontally and vertically with head-in-space rotation; in this situation the eye position calculated using calibration parameters determined for a particular head-in-space inclination (30° pitch, no roll) represents an approximation which accuracy decreases with the eye vertical eccentricity. To minimize this approximation, off-vertical angle was limited to 17°, so that the absolute head pitch actually varied between 13–47° and the absolute head roll between ±17°. In this range, the changes in initial eye position were shown to vary linearly horizontally by ∼5° and vertically by 5–10° (see figure 1–2 in [31]). We measured the error made by repeating the calibration procedures [36] for 5, 10 and 15° camera excursions at these extreme positions, and calculated a mean error of 5.3±0.84%. Because the vertical eye movements generated in present tests never exceeded 20° amplitude, measurements under these conditions have a confidence range close to 95%. Finally, no absolute zero position was defined such that horizontal and vertical signal processing consisted in a quantification of the relative change in eye position and velocity over time [37] which does not account for 2^nd^ order effects [18].

**Figure 2:**
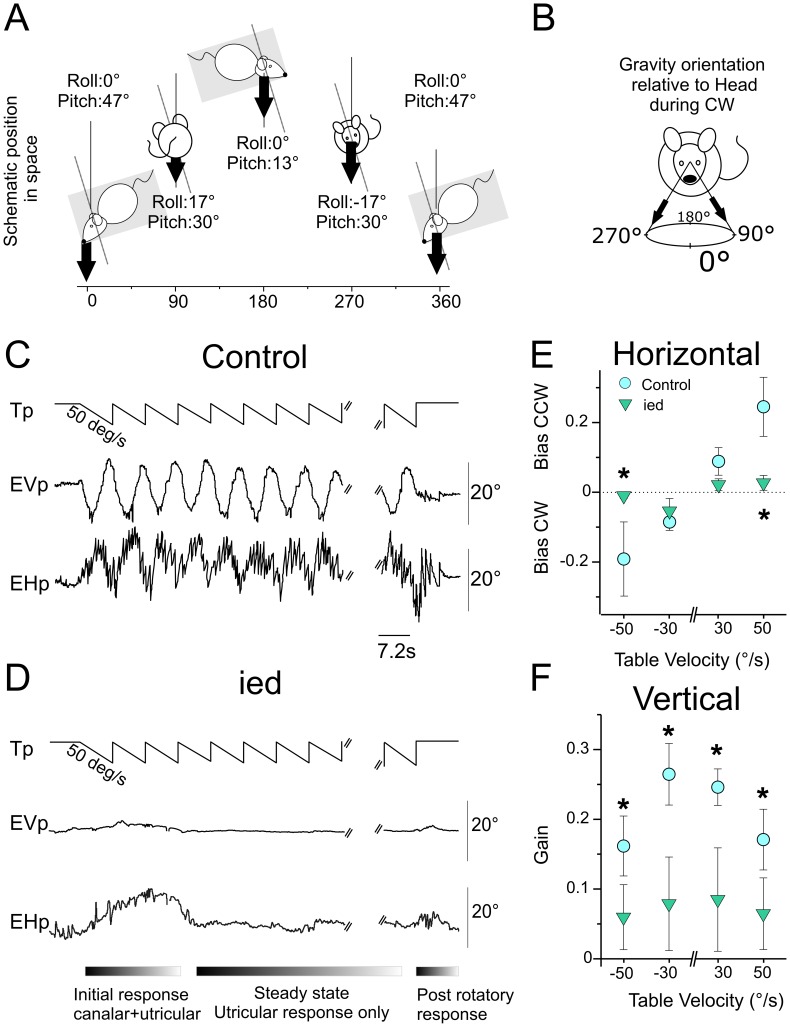
Maculo-ocular reflex in *C57Bl/6J* and *ied* mice. A, Left panel: Scheme of spatial displacement of the mouse during counter-clockwise rotation at constant velocity. Mouse was head-fixed 30° nose down; pitch of table was 17°. Roll and Pitch angles variations during rotations are reported for every ¼ cycle. B, Orientation of the gravity in head-fixed coordinates during clockwise rotation. C, Eye movements observed during 50°/s constant velocity off-vertical axis rotation in counter clockwise direction. Vertical eye position changed periodically with table rotation, reaching maximal elevation and depression when head roll was maximal (±8.5°). Horizontal eye movements also consisted in periodical modulation of the eye position on which was superimposed a horizontal nystagmus in compensatory direction (left for CW; right for CCW rotations). D, in *ied* mice, absence of otoconia resulted in the absence of horizontal and vertical movements during the steady-state. E, F, horizontal bias (E) and vertical gain (F) for *C57Bl/6J* and *ied* mice during off vertical axis in all tested conditions. In controls, horizontal bias increased with increasing table velocity. Vertical velocity gains decreased with increasing table velocity. Plots illustrate the absence of modulation of horizontal bias and vertical gain in *ied* mice. Asterisks indicate significantly larger response in *C57Bl/6J* compared to *ied* mice. Tp, Table position; EVp, Eye Vertical position; EHp, Eye Horizontal position.

### Data Analysis

Analysis procedures for horizontal angular vestibulo-ocular reflex (aVOR) have already been reported elsewhere [27]. Briefly, horizontal and vertical eye and head movement data were digitally low pass-filtered (cut-off frequency: 40 Hz), and position data were differentiated to obtain velocity traces. Segments of data with saccades were excluded from analysis. For horizontal sinusoidal rotations, at least 10 cycles were analyzed for each frequency. VOR gain and phase were determined by the least-squares optimization of the equation (a):
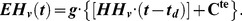
(a)where EHv(t) is eye horizontal velocity, g (gain) is constant value, HHv (t) is head horizontal velocity, td is the dynamic lag time (in msec) of the eye movement with respect to the head movement, and C^te^ is an offset. td was used to calculate the corresponding phase (ϕ°) of eye velocity relative to head velocity. The Variance-Accounted-For (VAF) of each fit was computed as



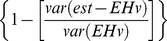
where *var* represents variance, *est* represents the modeled eye velocity, and *EHv* represents the actual eye horizontal velocity. VAF values were typically between 0.70–1, where a VAF of 1 indicates a perfect fit to the data. Trials for which the VAF was less than 0.5 were excluded from the analysis.

To analyze the oculomotor fields, vertical position was plotted as a function of horizontal position. The distribution of horizontal and vertical eye positions were then computed as an ellipse with half-axis set at 1.96*standard deviation (ellipse therefore represents 95% confidence interval of the distribution). Inclination of the ellipse was set as the slope of the linear regression between vertical and horizontal eye positions.

Time constant of the aVOR was measured in response to angular rotation at constant velocity (50°/s). The horizontal slow phase velocity decay was fitted to an exponential curve (f(x) = a*exp(b*x)) using Matlab cftool function and the time constant τ was then calculated as τ = −1/b.

To analyze maculo-ocular reflex (MOR), horizontal and vertical eye movements recorded during OVAR were processed separately. For horizontal OVAR responses, quick-phases were identified and removed [32]. During rotations, the velocity of horizontal slow phases is modulated (modulation, μ) around a constant bias (β). Both parameters (μ and β) were calculated from the sinusoidal fit of eye horizontal slow-phase velocity using the least-squares optimization of the equation (b):
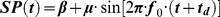
(b)where SP(t) is slow-phase velocity, β is the steady-state bias slow phase velocity, μ is the modulation of eye velocity, f_0_ is the frequency of table rotation.

To analyze vertical OVAR responses, the eye vertical position signal (EVp) was analyzed using a Fast Fourier Transform (equation (c)). Amplitude of vertical eye movements (EVa) was then calculated at table frequency (f_0_) (equation (d)). Eye velocity was computed following equation (e):
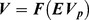
(c)

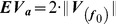
(d)

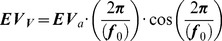
(e)


The gain of vertical eye movement was defined as the ratio between the eye velocity and the table velocity. The phase was calculated as the difference between the phase angles of the Fast Fourier Transform of eye and table signals as given by the Matlab angle function.

### Statistics

Statistical processing of all results was carried out using the Statistica 7.1 software (StatSoft France). Due to variable sampling size, influence of hypergravity (HG) on the VOR and MOR was tested by comparing *controls* (no HG) to developmental groups (*Pre*, *Post* and *Full*) and adults exposed to hypergravity (*Adult-HG*) groups using the non-parametric ANOVA Kruskal-wallis, followed by a post-hoc analysis. Comparison between *ied* and *controls* was achieved separately using the same procedure. For sake of clarity, the following text reports only the two-by-two post-hoc comparison between *controls* and each hypergravity-exposed group. All numbers presented in text and figures are mean ± standard deviation.

## Results

### VOR Responses in Controls *C57Bl/6J* versus Otoconia-deficient Mice

During horizontal sinusoidal rotation, aVOR consisted in *control* mice in horizontal eye movements and little vertical eye movements (Fig. 1A). In contrast, large vertical eye movements were observed in *ied* mice. Figure 1B shows in *ied* an example of the horizontal eye movements and of the abnormal vertical response evoked during 1 Hz aVOR. In *control* mice, the oculomotor fields could be represented as ellipses with a constant ratio between vertical and horizontal axis of about 0.15 at all frequencies (Fig. 1C). The inclination of the eye during sinusoidal movement (slope of the regression on Fig. 1A, B) was in range ±0.13, with a mean of −0.008±0.062. Figure 1C illustrates the regular relation between horizontal and vertical oculomotor fields across tested frequencies and the homogeneity of the aVOR responses among *control* mice. In *ied*, vertical eye fields were significantly larger than in controls (Fig. 1D; *p*<0.01 at all frequencies). Horizontal eye fields were also larger (*p*<0.019 at 1.5 and 2 Hz). The oculomotor fields of *ied* population therefore consisted in broader ellipses with a vertical/horizontal ratio in range 0.34 (at 1.5 Hz)–0.63 (at 0.2 Hz). Overall, slopes of the regression revealed that there was no consistency in the vertical responses observed in *ied* mice (range: −0.19– +0.7; mean 0.054±0.174; *p*>0.161 compared to controls at all frequencies). When only horizontal components were considered, the gain (p = 0.032) and the phase (p = 0.0052) of the horizontal aVOR were different compared to controls. Thus in general the horizontal aVOR in *ied* was qualitatively intact, but quantitatively impaired and perturbed by larger vertical eye displacements than observed in *controls*.

### Maculo-ocular Reflex (MOR) in *Controls* C57Bl/6J versus *Otoconia-deficient* Mice

The maculo-ocular reflex of mice was tested using constant velocity rotations with the table angled 17° off-vertical axis (Fig. 2A). In an egocentric frame of reference, off-vertical axis rotation (OVAR) makes the gravity vector continuously rotating around the head in a 17° wide circle (Fig. 2B). In *control* mice, OVAR evoked distinct horizontal and vertical responses similar to those previously described in other lateral-eye species (rabbit: [38]; rat: [18]; gerbil: [37]). Because initial response is produced by both canals- and otoliths-related signals, the first cycles were discarded from the analysis (Fig. 2C and 2D, bottom). During the steady-state utricular-only response, the horizontal eye velocity was modulated around a constant bias. Both bias and modulation increased with the speed of rotation. In *control* mice, 50°/s rotations in CCW direction evoked a mean gain bias of 0.24±0.08 and a mean gain modulation of 0.09±0.04. The corresponding phase was −40.3°±6.11. Vertical responses decreased with increasing table velocity. The gain of vertical eye velocity during 50°/s CCW rotations was 0.17±0.04. The mean corresponding phase was −0.93°±2.8. At the end of rotation, post-rotatory responses comparable to those observed following yaw rotations were observed.

In *ied* mice, onset of rotation was often associated to erratic eye movements (Fig. 2D). In the absence of utricular modulation and while constant velocity rotation continued, the eyes stabilized and remained almost immobile until the rotation was stopped. Post-rotatory canal responses were then always present. Absence of MOR was observed in all *ied* mice, during both CW and CCW rotations and at all tested velocities (Fig. 2E and 2F).

**Figure 3:**
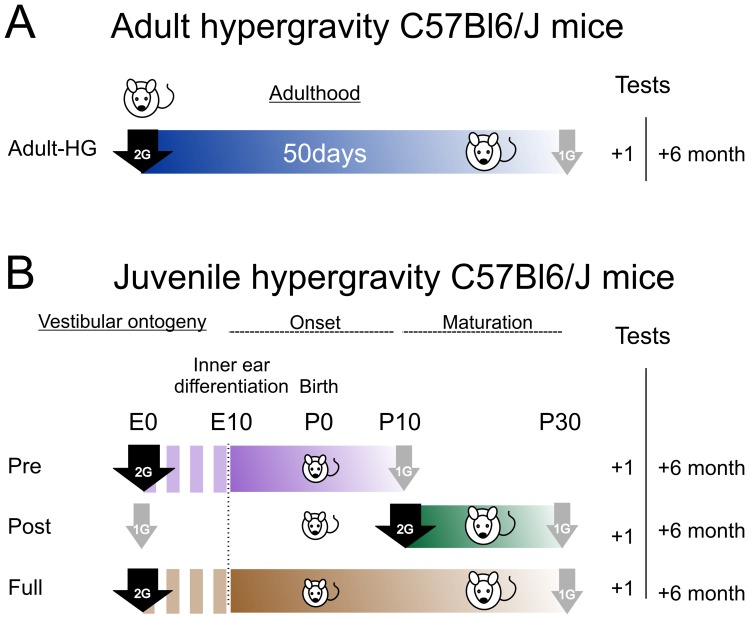
Scheme of hypergravity groups and timing of the experiments. A, *Adult-HG* group was composed of two month old mice centrifuged at 2G during 50 days. Vestibulo-ocular responses were tested 1 and 6 months after return to normal gravity. B, developmental groups. Mice were conceived, born and raised in 2G centrifuge (50 days, group *Full*), or exposed 20 days to hypergravity during early onset (*Pre*) or late maturation (*Post*) periods of vestibular development. For all groups, “+1” and “+6” refer to recording made 1 and 6 months after return to normal gravity, respectively.

*ied* mice demonstrate that in the absence of detection of gravity by the otoliths (as revealed by the absence of MOR), even reflexes based on semi-circular canals such as horizontal aVOR might be impaired. Would a prolonged change in gravity affect both macula-ocular and canal-related reflexes? How do VOR develop if the appropriate assembly of otoliths and semi-circular canals signals is perturbed? In order to evaluate these questions the vestibular responses of *C57Bl/6J* mice exposed to hypergravity at the adult stage and/or at different periods of their development were tested. Figure 3 summarizes the centrifugation protocols and the timing of the experiments.

### Long Term Effects of 50 Days of Exposure to Hypergravity in Adult *C57Bl/6J* Mice

To determine the effects of long term centrifugation on VOR, Adult *C57Bl/6J* adult mice were exposed to 50 days of 2G hypergravity (Fig. 3A). The aVOR and MOR were tested 1 and 6 months after the end of centrifugation.

**Figure 4:**
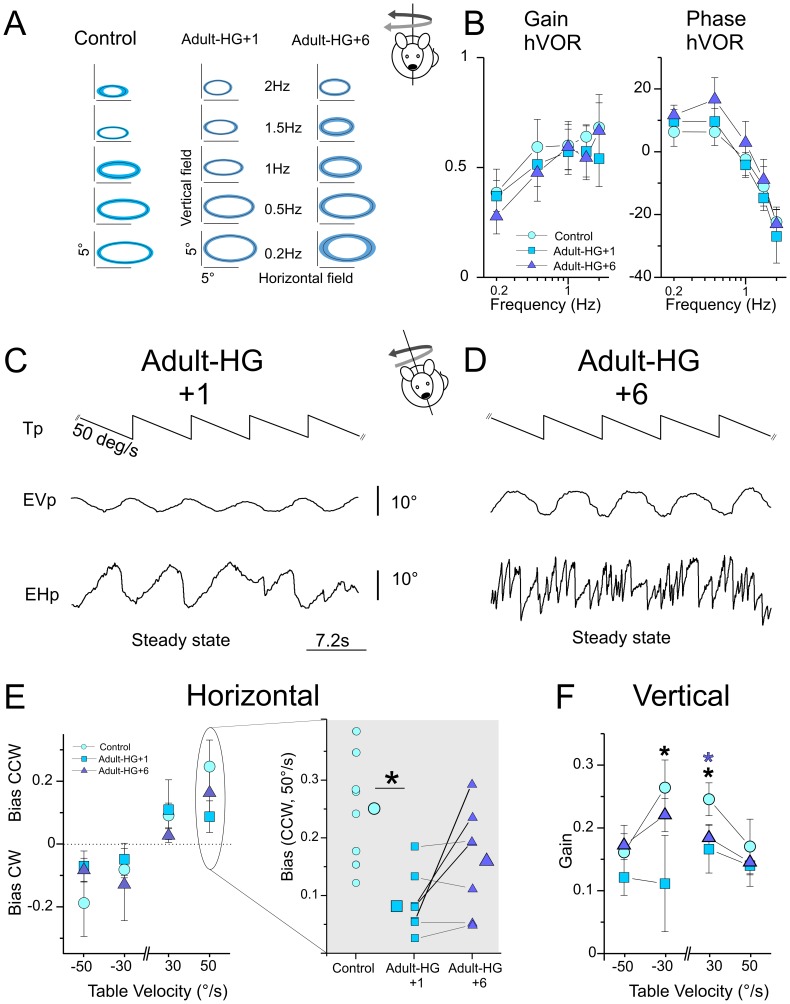
Modification of the angular vestibulo-ocular and maculo-ocular reflexes in adult centrifuged *C57Bl/6J* mice. A, Averaged oculomotor fields in non-centrifuged (*control*) and centrifuged (*Adult-HG)* mice across tested frequencies. Line and surface of the ellipses present the mean and standard deviation of the population, respectively. The slopes of the ellipses are the mean slope of the individuals’ ellipses. Oculomotor fields were not altered in *adult-HG* compared to *control*. B, Bode plots of horizontal VOR in dark gain and phase during horizontal sinusoidal rotations. There was no consistent effect of centrifugation on the responses to sinusoidal rotations. C–D, Raw traces showing steady state response of the same adult-HG mouse followed at time +1 month (C) and +6 months (D) after centrifugation. Note the absence of nystagmus on the horizontal traces and the strong reduction in the amplitude of the vertical movements at 1 month. E, horizontal bias (E) for non-centrifuged (*control*) and centrifuged (*Adult-HG)* mice in all tested conditions. Horizontal biases (E) were significantly affected in 50°/s CCW condition. Right panel presents individual (small symbols) and mean of the populations (large symbols). Solid lines indicate the evolution of individuals’ bias at +1 and +6 months after centrifugation (black: improved responses; dotted: no or little improvement in the responses). F, vertical gain (F) for non-centrifuged (*controls*) and centrifuged (*Adult-HG)* mice in all tested conditions. Tp, Table position; EVp, Eye Vertical position; EHp, Eye Horizontal position. Black or purple asterisks indicate *p*<0.05 between *control* and *Adult-HG+1 or Adult-HG+6,* respectively.

When tested with sinusoidal rotations the aVOR of centrifuged Adults-HG was normal compared to non-centrifuged adults (n = 8). First, oculomotor fields revealed no difference in the horizontal/vertical ratios as observed in the otoconia-deficient mice (Fig. 4A). In addition, both the gain and phase of horizontal aVOR were unchanged at all tested frequencies (up to 2 Hz; Fig. 4B).

The MOR of Adults-HG was tested during OVAR stimulation (n = 7). As illustrated on figure 4C, the eye movements evoked in Adults-HG were strongly impaired. In particular, the horizontal compensatory nystagmus was found to be reduced or even absent. The modulation of horizontal eye position was periodic and followed cycles of rotations resulting in uncompensatory displacements (e.g. presence of CW directed slow-phases during CCW rotation). Furthermore, both horizontal and vertical modulations of the eye position were reduced in amplitude (compare traces in Figure 2C and 4C; mean vertical amplitude at 30°/s: 10.2°±2.85 in centrifuged adults vs. 17.0°±2.25 in controls). Horizontal gain bias was reduced 1 month after the end of centrifugation to 0.08±0.05 compared to 0.24±0.08 in controls (p<0.001; post-hoc analysis CW 50°/s, p = 0.042; CCW 50°/s rotations *p*<0.001; Fig. 4E). Vertical gains were also significantly reduced (Fig. 4F: p<0.001; post-hoc analysis CCW 30°/s rotations *p* = 0.011; CW 30°/s rotations *p*<0.001). Taken together, MOR responses were diminished by a factor 2 to 3 compared to control conditions. To investigate the persistence of these deficits, we recorded again the VOR and MOR 6 months after the adult mice returned to normo-gravity.

The responses to sinusoidal rotations and to yaw constant rotations remained normal during the 6 months following the end of the centrifugation (Fig. 4A and B). There was an improvement in the MOR during the same period. Figure 4D shows the evolution of the eye movements observed in the same animal as in Figure 4C. At 6 months, the horizontal eye movements were compensatory, although the nystagmus was often less pronounced than in controls. The mean horizontal gain bias of adult-HG improved (see right panel in Figure 4E). Recovery of MOR was quite heterogeneous between individuals (Fig. 4E, right panel). While the horizontal responses (thick lines) improved in some animals and reached the range of control values, other animals showed no or little improvement (thin lines). Overall, adult-HG MOR appeared strongly modified 1 month after the end of the centrifugation. During the following months, a substantial readaptation to normal gravity was observed, however we note that the quality of the recovery was quite heterogeneous among individuals.

In adult mice, 50 days of hypergravity altered durably the MOR, but not the horizontal aVOR. To assess how a change in gravity would affect the development and maturation of vestibulo-ocular circuitry, we then tested animals which developed in the centrifuge.

### Effects of Hypergravity on the Acquisition of aVOR

To assess the developmental effects hypergravity, *C57Bl/6J* mice were raised under 2G during specific periods of development (Groups Pre and Post; fig. 3B) or entire development (group full).

**Figure 5:**
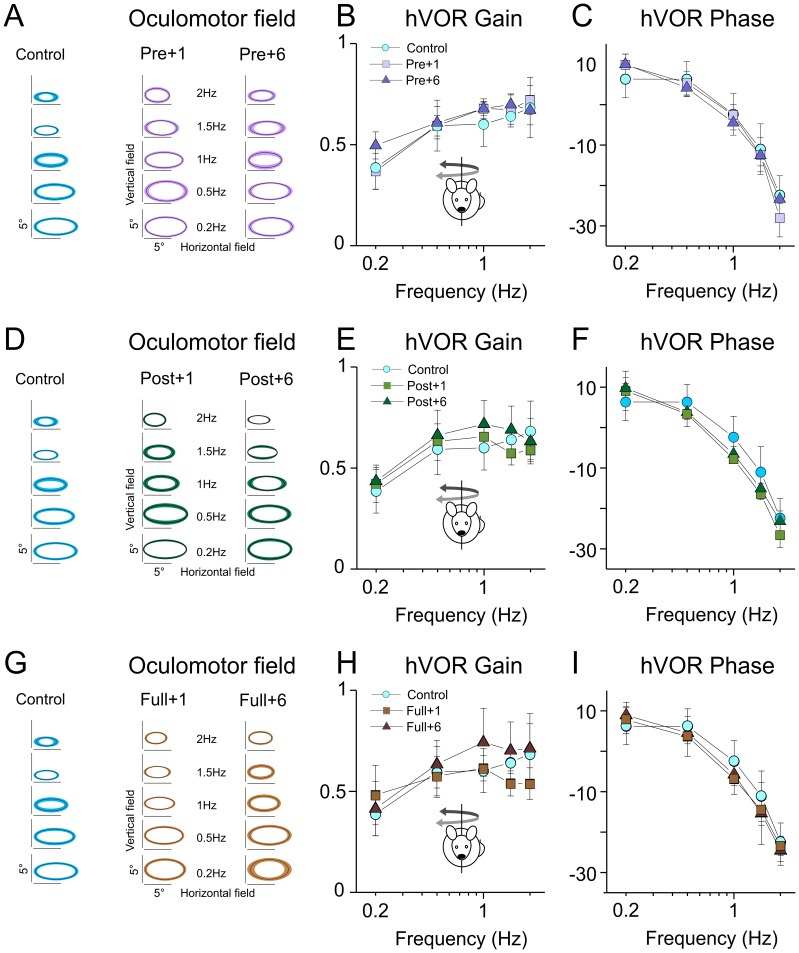
Angular vestibulo-ocular reflex is not modified following developmental exposure to hypergravity. A, B, C: Averaged oculomotor fields (A), horizontal gain (B) and phase (C) in non-centrifuged (*control*) mice compared to mice centrifuged between E0–P10 (*pre*) tested 1 and 6 months after centrifugation. No significant differences were found. D, E, F: Averaged oculomotor fields (D), horizontal gain (E) and phase (F) in non-centrifuged (*control*) mice compared to mice centrifuged between P10–P30 (*post*) tested 1 and 6 months after centrifugation. No significant differences were found. G, H, I: Averaged oculomotor fields (G), horizontal gain (H) and phase (I) in non-centrifuged (*control*) mice compared to mice centrifuged between E0–P30 (*full*) tested 1 and 6 months after centrifugation. No significant differences were found.

In groups *Pre* and *Post* centrifuged for only part of their development, or in group Full, no significant alterations were found during sinusoidal testing. Figure 5A, D, G present the oculomotor fields of the different populations of mice tested 1 and 6 months after end of centrifugation. No abnormal vertical movement was observed in any of the groups. In addition, both the gain (Fig. 5B, E, H) and phase (Fig. 5C, F, I) of the aVOR was normal compared to controls in all conditions. These results suggest that, unlike what was observed in otoconia-deficient mice, alteration of gravity during the development does not impair the spatial tuning of canal-related reflexes.

### Effects of Hypergravity on the Acquisition of Maculo-ocular Reflex

**Figure 6:**
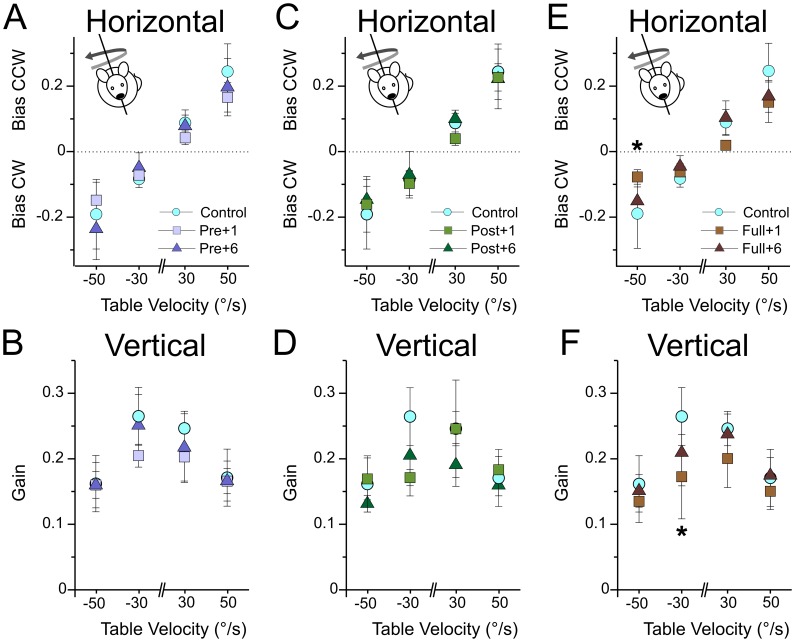
Maculo-ocular reflex in mice centrifuged during development. A–B, horizontal bias (A) and vertical gain (B) for *control* and *pre* mice during off vertical axis rotation in all tested conditions. No significant differences were found at 1 and 6 months. C–D, horizontal bias (C) and vertical gain (D) for *control* and *post* mice during off vertical axis rotation in all tested conditions. No significant differences were found at 1 and 6 months. E–F, horizontal bias (E) and vertical gain (F) for *control* and *full* mice during off vertical axis rotation in all tested conditions. Asterisks indicate significant differences (p<0.05) between *control* and *full+1* groups.

Does exposure to hypergravity during development induce long term alteration of MOR as observed in the adults after fifty days of hypergravity? One month after end of the centrifugation, we found no statistical differences in the OVAR responses of the *Pre* and the *Post* groups compared to controls. No significant change was observed on the eye horizontal component (Fig. 6A, C) or in the vertical component (Fig. 6B, D).

These results demonstrate that 3 weeks exposure to hypergravity before or after P10 had no measurable effect on the MOR. What happens when hypergravity covers the entire development? Again, we found no major difference in the MOR responses of the *Full* group. Extending hypergravity exposure to the entire development however had significant effects (Fig. 6E, 6F), but the variability in the responses was quite large. The reduction in vertical eye movements was comparable to the one observed for *Adult-HG*; however the horizontal eye movements were both qualitatively and quantitatively better, i.e. compensatory nystagmus was present and bias values were greater in *Full* group than in *Adult-HG*.

How did the MOR of the developmental groups evolved during the following months? As expected, both *Pre* and *Post* groups presented completely normal MOR (Fig. 6A–D). The group *Full* had recovered normal responses: vertical eye movements were not different from controls. For horizontal eye movements, improvement in the bias was observed in previously affected conditions and henceforth comparable to *controls* in all conditions.

These observations suggest that centrifugation during entire, but not specific parts, of development might have transitorily delayed acquisition of normal MOR. However after return to normal gravity, the juvenile mice show no long term impairment so that 2G hypergravity did not reveal any critical period during VOR ontogeny.

## Discussion

### Otoconial Agenesis Affects Vestibular Signal Processing and Gaze Stabilization

How does complete absence of otolith-related inputs affect vestibular processing? Observations made on different otoconia-deficient mice showed that the absence of modulation of the macular signals was detrimental for the development of vestibulo-motor circuitry. In the *tilted* mice, another otoconia-deficient strain, the impairment in aVOR was functionally compensated by an increase in the efficiency of the optokinetic reflex [39]. Our observation that many *ied* mice initially suffer from ocular instability (Figure S1) when put in dark also suggests that, in the absence of otolith information, visual inputs become instrumental for gaze stabilization. The most striking deficit in *ied* was that horizontal head movements triggered abnormal vertical eye movements. What is the origin of this impairment in the spatial tuning of aVOR? .

We have checked experimentally that the deficit was not due to a misalignment of the semi-circular canals during the rotation (Figure S1). The deficit could alternatively originate from the abnormal branching at the level of the semi-circular canals. For instance, otoconial agenesis could result in a mis-directed innervation of the cristae by otolith afferent axons during the development of the inner ear. However, no morphological or cytological abnormalities were reported in the cristae ampullares of *ied* mutants [20]. Similar observations were made in *het* [40] and *tilted* strain [21]. Most of the studies which investigated vestibular periphery or afferents in otoconia-deficient strains instead concluded that absence of modulation in the macular afferents was the main consequence of the mutation. Hence, it was demonstrated in *Het* mice that presence of otoconia was not required for the general formation and maintenance of synapses [41] or normal development of vestibular ganglia [42]. The resting discharge pattern of macular primary afferents was also unperturbed in otoconia-deficient mice (*Head-tilt* and *tilted*; [19]). Further characterization of the innervations and physiological properties of the semi-circular canals and afferents in *ied* would however be required to exclude the peripheral origin of the observed deficit.

Another hypothesis would be that the central branching or processing of vestibular information at the level of vestibular nuclei is responsible for the default in spatial tuning of aVOR. Ontogeny of central vestibulo-ocular circuitry depends initially on genetic [6]. Activity-mediated spatial refinements of connections and fine tuning of the temporal dynamics of aVOR occurs during a second developmental phase [9], [10]. It has been suggested in larval frog that otolith-driven responses in vestibular circuits could serve as a spatial reference frame for the refinement of canal-driven responses during early development [43]. Present results are compatible with this hypothesis, such that in the absence of gravity-related signals, central vestibular neurons would substitute otolith inputs with spatially non-matching canal inputs. Comparable process was suggested in labyrinthectomized frogs as a “basic reaction pattern” which substituted the missing utricular inputs with commissural and/or afferent signals. Functionally, the restoration of macula-ocular reflex gain also led to the impairment of its spatial-tuning [44].

At this stage, the peripheral and/or central origin of the deficit in the spatial tuning of aVOR observed in *ied* remains uncertain. Characterization of the electrophysiological responses in canal afferents and in central vestibular neurons would be required to sort out the cause for the abnormal eye movements. In addition, the same experimental paradigm could be applied on other otoconial mutant to confirm that it is the absence of modulation of the otolith-related signals which is the origin of this deficit and therefore exclude some other central neurologic ramifications.

### Hypergravity Transiently Affects Macula-ocular Reflex in Juvenile and Adult Centrifuged Mice

How are vestibular organs and central integration of multisensory inputs affected by altered gravity? On ground, an alteration of gravity can be produced by mean of centrifuges [25]. Prenatal exposure of rats to hypergravity have been reported to affect sensory epithelium morphology [45], local connectivity [46], physiology of hair cells [13], glutamatergic neurotransmission between hair cell and afferent neuron [47] and peripheral vestibulocerebellar afferents [48]. Bouët and collaborators [14], [15], [49] reported in rats that development and maturation under 2G hypergravity induces postural and locomotor deficits which all recovered in about 3 weeks. There is currently little evidence of a permanent deficit following development under hypergravity. Sondag et al. [17] however showed that a 20 weeks long development of hamsters at 2.5G induces inabilities in swimming and air-righting reflexes even 8 months after return to normal gravity. Overall, an increase in gravity-load during development affects both peripheral and central vestibular-related circuits. Most of the changes however seem transitory and highly depend on the level of gravity-load imposed and on the length of exposure to HG.

Present results on VOR extend these previous experiments. We have shown that a 3 week long exposure to hypergravity during development does not alter the acquisition of vestibulo-ocular reflex in *Pre* and *Post* groups. However, a 50 days exposure of juvenile mice (*Full* group) induced a delay in the acquisition of macula-ocular reflex. This retard is compatible with a delayed development of the vestibular periphery [13], [45]–[47]. Another hypothesis could lie in the central integration of canal and otolith inputs: in particular, the central process known as “velocity storage” which provides a spatially referenced estimate of head velocity [50], [51] could be affected. Velocity storage normally allows compensating the deficiencies of peripheral vestibular sensors to accurately estimate rotation velocity, and to distinguish between a prolonged linear acceleration and a head tilt [52]–[54]. Shortening of aVOR time constant was observed in weightlessness [55], [56] or after sustained centrifugation [57] and is attributed to a decreased coupling between canalar and otolith inputs [57], [58]. The impairment we observed in the horizontal bias during OVAR is compatible with this hypothesis. More experiments will however be needed to determine how a 50 days long exposure to hypergravity affects the velocity storage mechanism.

Adult-HG mice presented the most striking and persistent alteration of macula-ocular reflex following exposure to hypergravity. In the absence of histological analysis of the effects of the prolonged centrifugation on the inner ear structures, we cannot exclude that the observed persistent alteration could reflect damage to the inner ears as a result of the chronic centrifugation. However, previous experiments on rats suggested that the vestibular sensory epithelium was qualitatively unaffected after a prolonged period of hypergravity [11], [17]. The deficits in adult-HG mice could alternatively reflect the difficulty of mature brains to adapt/readapt to successive alterations in gravity. As in other sensory systems, the plastic processes underlying adaptation to gravitational changes appear variable among individuals and more restricted in mature brain than during development [59].

### A Vestibular Critical Period during Ontogeny of Motor Control?

The existence of a critical period during which the vestibular information are mandatory for the motor development is disputed [60]. It is supported by the permanent inability to swim and to perform surface righting in rats that matured in space [61], [62], and by the permanent motor deficits in mice centrifuged between P10–P30 [25], [26]. In rodents, deprivation experiments claim for an early need for vestibular inputs. The complete removal of vestibular organs in rats before P5 leads to permanent head-bobbing in adults [63], [64], and mice which congenitically lack all vestibular organs show a typical shaker/waltzer behaviour, head-bobbing and abnormal locomotion [29], [65].

Interestingly, specific unilateral lesion of the otolith in the adult guinea-pig [66] triggered a transient head-tilt and postural symptoms, which resembled those observed in otoconia-deficient mice. These symptoms markedly differed from the plane specific horizontal oscillations and circling observed after unilateral lesion of the horizontal canal [63] or unilateral vertical canal lesions [66]. These postural syndromes observed after selective vestibular impairment would suggest that the permanent head-bobbing and circling in vestibular-deprived mutants could reflect a critical need for the information coded by the vertical and horizontal semicircular canals, respectively, rather than by the otoliths. On the other hand the permanent oculomotor deficits we describe here in *ied* and the permanent head-tilt observed in otoconia-deficient strains could be the signature of an otolithical critical period.

The environmental removal of gravity during development is another way to test the hypothesis of an otolith-specific critical period. In rodents, transient changes in gravity have been tested by exposing animal to weightlessness (µG) during space journey. Experiments conducted on rats exposed to environmental deprivation of gravity during gestation concluded in a normal development in space. Hence, no change were found on ground in motor behavior such as walking (11 days µG between embryonic day E9–E20; [67]), righting response, negative geotaxis or rotating platform (5 days µG, E13–E18; [68], [69]). However, following 11 days in µG (E9–E20) vestibular-specific tests suggested a retarded ability to respond to gravistatic stimuli [70], abnormal water immersion responses [71], and increased bradychardia following roll stimulation [7].

In conclusion, genetic suppression of gravity-related signals indicated that otolith-related signals might be necessary to ensure a proper functioning of canal-related vestibular reflexes. It also suggested an otolithical critical period for motor control, which could be the pendant of a canalar critical period previously described. On the other hand, exposure to hypergravity during development was not sufficient to alter durably motor behaviour. However, in centrifuge the sensing of gravity is altered rather than suppressed as it would be during a space journey. More experiments with long (>2 month) exposure of mice to weightlessness will be needed to rule out the existence of critical period during vestibular development.

## Supporting Information

Figure S1 **Spontaneous oculomotor instability and determination of head pitch in otoconia-deficient mice.** A, Spontaneous ocular instabilities in horizontal, vertical and torsion (not shown) were observed. Nystagmus was present in dark (left), diminished at light (center) and abated as the animal got accustomed to the apparatus (right panel). B, Gain of the horizontal VOR at light measured at different head pitch angle. Gain was found to be maximal at 30°. Note that oculomotor fields horizontal and vertical components did not vary according to head pitch, suggesting that the deficit observed in *ied* was not related to a misalignement of the semi-circular canals.
(TIF)
