# Supplementary figures and images for: Ontogeny of Mouse Vestibulo-Ocular Reflex Following Genetic or Environmental Alteration of Gravity Sensing
Source: PLoS One. 2012 Jul 10;7(7):e40414. doi: 10.1371/journal.pone.0040414 (PMC3393735; doi:10.1371/journal.pone.0040414)

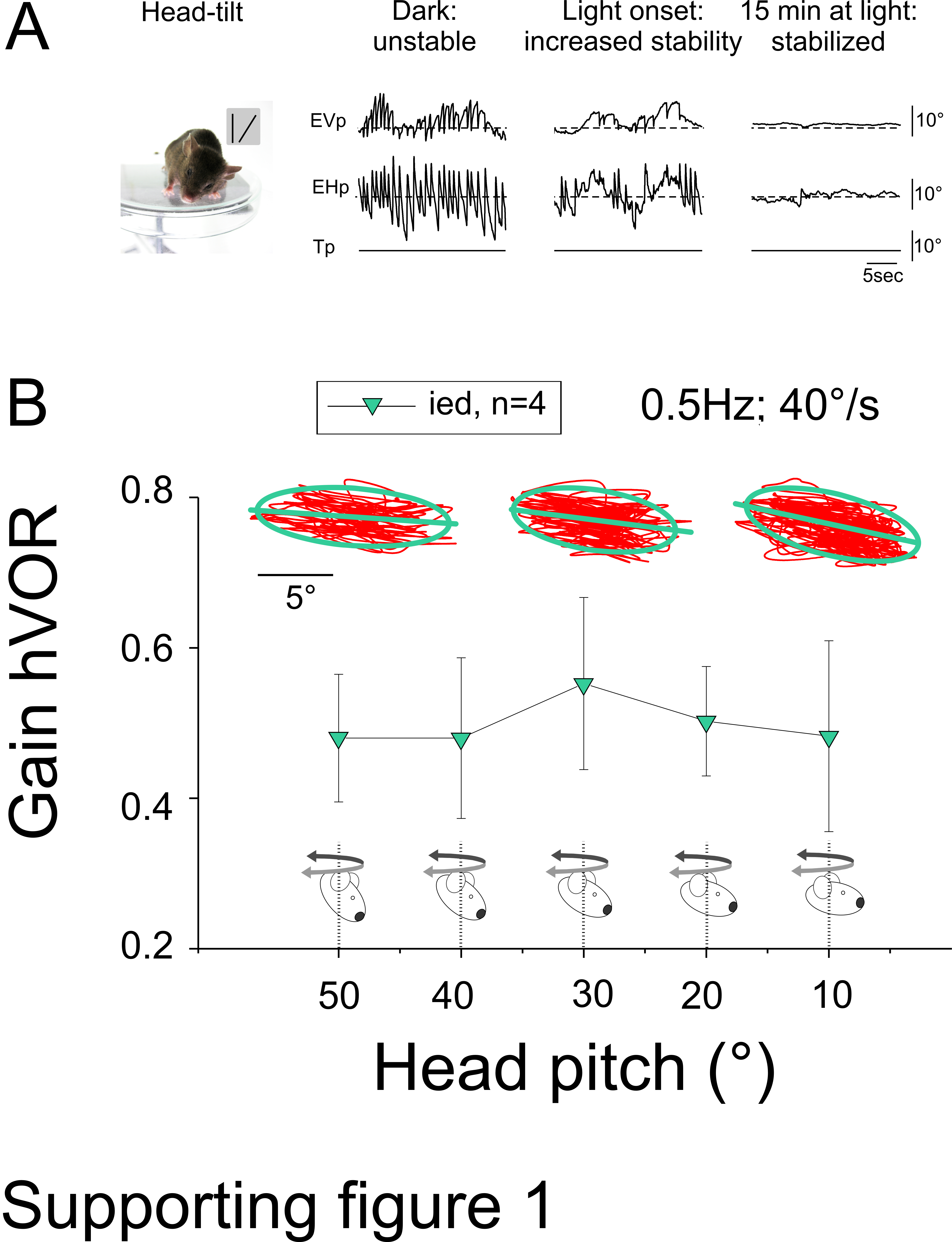

Supplement: Figure S1 — Spontaneous oculomotor instability and determination of head pitch in otoconia-deficient mice. A, Spontaneous ocular instabilities in horizontal, vertical and torsion (not shown) were observed. Nystagmus was present in dark (left), diminished at light (center) and abated as the animal got accustomed to the apparatus (right panel). B, Gain of the horizontal VOR at light measured at different head pitch angle. Gain was found to be maximal at 30°. Note that oculomotor fields horizontal and vertical components did not vary according to head pitch, suggesting that the deficit observed in ied was not related to a misalignement of the semi-circular canals. (TIF) [file pone.0040414.s001.tif]
